# Physical Exercise as Personalized Medicine for Dementia Prevention?

**DOI:** 10.3389/fphys.2019.00672

**Published:** 2019-05-29

**Authors:** Patrick Müllers, Marco Taubert, Notger G. Müller

**Affiliations:** ^1^ Neuroprotection Laboratory, German Center for Neurodegenerative Diseases (DZNE), Magdeburg, Germany; ^2^ Institute of Sport Science, Otto-von-Guericke University Magdeburg, Magdeburg, Germany; ^3^ Center for Behavioral Brain Sciences (CBBS), Magdeburg, Germany; ^4^ Medical Faculty, Clinic for Neurology, Otto-von-Guericke University Magdeburg, Magdeburg, Germany

**Keywords:** exercise, dementia, neuroplasticity, personalized medicine, responder

## Abstract

Accumulating evidence mainly from observational studies supports the notion that lifestyle factors such as regular physical activity can modulate potential risk factors of dementia. Regarding a potential mechanism for this interaction, results from intervention studies show that exercising can induce neuroplastic changes in the human brain. However, a detailed look at the study results reveals a wide interindividual variability in the observed effects. This heterogeneity may originate from the fact that there are “responders” and “non-responders” with respect to the impact of physical exercise on physiological outcome parameters (i.e., VO_2_ peak) and the brain. From this, it follows that recommendations for physical exercise programs should not follow a “one size fits all” approach. Instead, we propose that the exercises should be tailored to an individual in order to maximize the potential neuroplastic and preventive effects of regular exercise. These adaptations should take the individual performance levels into account and impact both the quality (i.e., type) and the quantity of exercises (i.e., intensity, duration, and volume).

## Introduction

According to recent predictions, the global number of people affected by dementia will rise from currently 47 to 131.3 million by 2050 (Prince et al., 2015), whereby Alzheimer’s disease (AD) as the most common cause of dementia accounts for up to 75% of cases (Masters et al., 2015). Hope for the imminent development of disease-modifying drug therapies has faded after more than 200 clinical trials with new drugs have failed in the recent past (Schneider et al., 2014). In this context, concepts of healthy aging are becoming increasingly important.

Due to the lack of prospect for causal pharmacological treatments, dementia research is currently directed toward modifiable risk and lifestyle factors serving as preventive strategies (Kivipelto et al., 2018a). Norton et al. (2014) postulate that one third of the global prevalence of Alzheimer’s disease is related to modifiable risk factors. Among other factors especially physical inactivity, overweight, hypertension, and diabetes mellitus have been identified as modifiable risk factors. The latter open an opportunity for various preventive strategies. According to a computational model, a 10% reduction of the risk factors per decade could lead to a decrease in 8.3% of the global Alzheimer’s prevalence by 2050 (Norton et al., 2014). Additionally, delaying the onset of dementia by 5 years could reduce the number of affected people by nearly 50% (Sperling et al., 2011) and would have a key public health impact.

Systematic reviews on epidemiological studies suggest a strong impact of regular physical activity (as opposed to mainly sedentary behavior) on dementia risk (Hamer and Chida, 2009; Sofi et al., 2011). For example, Hamer and Chida (2009) have shown in a meta-analysis including 16 prospective studies with 163,797 non-demented participants in which physical activity is associated with a reduced risk of dementia of all types of 26% and a reduced Alzheimer’s disease risk of 45%.

However, randomized controlled interventions reported mixed findings regarding the effect of exercising on cognition and the brain, casting some doubt on its preventive power against dementia (Müller et al., 2017). In summary, current research indicates that interventions are more beneficial at preclinical and early clinical stages of Alzheimer’s disease (Forbes et al., 2015; Brini et al., 2018). Regarding the multifactorial etiology of most dementia cases, current large multidomain trials [MAPT (Andrieu et al., 2017), PreDIVA (van Charante et al., 2016), and FINGER (Ngandu et al., 2015)] investigate the effect of lifestyle interventions on cognitive functions and, ultimately, dementia prevention. So far only the FINGER trial has revealed beneficial intervention effects on cognitive functions among participants at risk of dementia (Ngandu et al., 2015). Whether these effects have an impact on the later incidence of dementia in the participants is not yet clear. Actually, the global initiative *World Wide Fingers* aims to advance dementia prevention studies (Kivipelto et al., 2018b).

## Individual Response to Physical Exercise

Numerous epidemiological, cross-sectional, and interventional studies indicate that regular physical activity has positive effects on health in general and brain health in particular and has the potential to reduce the risk of dementia (Hamer and Chida, 2009; Sofi et al., 2011; Brini et al., 2018; Liu-Ambrose et al., 2018). However, a detailed analysis of the studies often reveals a wide interindividual variability of the results (Müller et al., 2018). The individual response to physical exercise has received attention in sport science since the 1980s (Rankinen and Bouchard, 2008). Especially in the context of endurance and strength training, there is strong evidence for different individual physiological adaptations to identical exercise and training variables (Buford et al., 2013; Weatherwax et al., 2016). Based on these wide interindividual variations, humans have been divided into “responders” or “non-responders” with respect to a specific exercise (Buford et al., 2013). Here “responders” are defined as subjects who achieve a benefit, while “non-responders” may exhibit an unchanged or even worsened performance under the same stimulus (Bouchard and Rankinen, 2001). However, the term “responder” is currently under discussion. For example, Booth and Laye (2010) proposed that the term “non-responding” should be replaced by the term “low sensitivity” accounting for the fact that usually so-called non-responders show some training effects after all albeit to a lower extent, indicating that they may “convert” to responders if the training is adapted for them, i.e., by increasing training frequency.

## Physical Exercise as Personalized Medicine

Personalized medicine is an approach for pharmacological drug treatment and preventive interventions based on individual variability in genetics, anthropometrics, biomarkers, environment, and other factors. Thereby, the terms “personalized medicine,” “precision medicine,” and “individualized medicine” are often used synonym. Especially in oncology, personalized therapies have been successfully used for years (Shin et al., 2017). Currently, this approach has become popular in dementia research as well, e.g., Hampel et al. (2017).

Referring to the observation of the interindividual variety in physiological adaptions in response to physical exercise from sport science, the concept of “responders” and “non-responders” would have fundamental implications on the proposed neuroprotective and preventive factors of physical activity on dementia, too. In this regard, the following questions arise: (1) Which factors cause the large interindividual heterogeneity in response to physical training? (2) Are all outcomes affected equally by the individual responsiveness? and (3) How can we overcome non-responsiveness so that (almost) all individuals experience benefits? In the following, we will briefly discuss these questions.

1. Which factors cause this large interindividual heterogeneity in response to physical training?
  Like with the risk for dementia (and other diseases), the individual physiological response to physical exercise is modulated by concomitant modifiable (e.g., diet) and non-modifiable factors (e.g., genetics, gender; Bouchard and Rankinen, 2001; Rankinen and Bouchard, 2008; Booth and Laye, 2010; Sparks, 2017). Regarding the latter, as of now over 150 genetic markers have been associated with elite athlete status (Ahmetov et al., 2016) and trainings response (Bray et al., 2009). Additionally, several single-nucleotide polymorphisms were identified as being related to the training response. Results of the HERITAGE (HEalth, RIsk factors, exercise Training And Genetics) study (Bouchard et al., 1999; Timmons et al., 2010) indicate that the interindividual variation in physiological responses to exercise is based, among other causes, on genetic factors. Here, 21 single nucleotide polymorphisms accounted for 49% of VO_2_ peak variation (Bouchard et al., 2011). Interestingly, VO_2_ peak has been associated with brain function in older adults (Erickson et al., 2009, 2011). Timmons et al. (2010) suggested molecular classifications based on 29 RNA signatures that predicted VO_2_ peak, whereby 11 single-nucleotide polymorphisms explain 23% of the variance in VO_2_ peak. However, only one intervention study has used a genetic-based algorithm for personalized resistance training (Jones et al., 2016). Altogether, several aspects of the connection between genotype and exercise response are still unclear (Mann et al., 2014).
2. Are all outcomes affected equally by individual responsiveness status?
  Both resistance and endurance trainings have yielded strongly varying results across individuals and, moreover, within an individual, the results are often inconsistent across different variables, so that the same person may present training induced benefits in one domain but not the other (Vellers et al., 2018). For example, after a 12-week resistance training, Hubal et al. (2005) reported that on average muscle size and strength increased in young adults. However, a closer look reveals that the gains in muscle size varied from −2 to +59% and those in strength between −32 and 149%. Similar results were reported following endurance interventions (Bouchard et al., 1999; Bouchard and Rankinen, 2001). Moreover, Karavirta et al. (2011) observed a wide range of individual responses to a combined endurance and strength training in older adults. After the 21 weeks of intervention, cardiorespiratory fitness (VO_2_ peak) gains varied from −8 to 42% and strength (maximal isometric bilateral leg extension) from −12 to 87%. Other trials have shown a similar interindividual heterogeneity regarding the VO_2_ peak response to exercise training in young (Kohrt et al., 1991; Bouchard and Rankinen, 2001) and old adults (Chmelo et al., 2015; Ross et al., 2015). Furthermore, other cardiorespiratory (e.g., blood pressure, heart rate at work load) and metabolic (e.g., insulin sensitivity, cholesterol) parameters have also shown strong interindividual differences in adaptation to exercise (Bouchard and Rankinen, 2001; Fritz et al., 2006). This interindividual variability could have a fundamental influence on the effect of physical exercise on neuroprotection and prevention of dementia, because especially cardiorespiratory and metabolic parameters are high-risk factors of dementia. A non-response to physical exercise on these risk factors could thus avert the positive effects of physical exercise on dementia risk in general. Other aspects such as gender (Barha et al., 2017a) or APOE (Berkowitz et al., 2018) are likely to influence the effects of physical activity.
  Thereby, cardiovascular, neuromuscular, and balance training improve differently cognitive performance and neuroplasticity in elderly (Voelcker-Rehage et al., 2011; Voelcker-Rehage and Niemann, 2013; Levin et al., 2017). There are only a few studies regarding interindividual variability following physical exercise interventions on cognitive functions. Heisz et al. (2017) reported that a 6-week exercise, cognitive, or combined training led to general improvements in memory functions in young adults. In more detail, individuals with greater cardiovascular improvements had also larger increases in levels of the brain-derived neurotrophic factor (BDNF) and the insulin-like growth factor-1 (IGF-1). Furthermore, high responders to exercise in the combined training group had better memory performance compared with exercise alone.
3. How can we account for the interindividual heterogeneity to achieve optimal results in (almost) all individuals?
  Following the hypothesis that there is a clinical relevant group of “non-responders” or “low sensitivity,” the question raises whether modifications of a given exercise program (e.g., type of exercise, exercise durations, exercise volume, exercise intensity) can overcome the lack of training effects. Some current studies indicate that the non-responder status can be mitigated by increasing the exercise intensity and/or dose (Bonafiglia et al., 2016; Lundby et al., 2017; Montero and Lundby, 2017). For example, Montero and Lundby have shown that the percentage of non-responders is lower when the training is accomplished four to five times per week as opposed to only one to two times. Regarding effects on cognitive function, the optimal dose-response relationship is still largely unknown. One potential mediator of training effects on brain function is BDNF, which enhances neuroplasticity *via* different pathways (Brigadski and Leßmann, 2014). BDNF excretion is induced by lactate (Schiffer et al., 2011), and peripheral blood lactate levels have also been shown to correlate with cognitive improvements (Lee et al., 2014; Tsukamoto et al., 2016). Consequently, exercise interventions should be intensive enough to increase lactate and, as a consequence, BDNF. However, especially in older adults, classical exercise interventions rarely achieve the second ventilatory threshold (VT2) level, which is associated with an accumulation of lactate. Using High Intensity Interval Training (HIIT) with higher intensities and lower volumes could be a method to achieve higher numbers of responders. Considerable evidence is accumulating regarding the positive effects and safety of HIIT training strategies even for older adults and patients with chronic diseases (Ross et al., 2016) (e.g., chronic heart failure, COPD, diabetes).

## Future Recommendations

Actually, personalized medicine is primary concerned with heterogeneity in an individual’s response to medical drugs. But, there is also an urgent need for personalized preventive exercise strategies (Buford et al., 2013; Berkowitz et al., 2018). Thereby, personalized exercise programs would enhance training efficiency and improve more outcome variables in a larger number of individuals. For future intervention studies, genetic analyses could help to identify potential “responders” and “non-responders.” Furthermore, a personalized exercise training program should be based on a complex performance analysis, especially to identify specific individual weaknesses. More research is needed to provide more detailed recommendations for the suggested personalized exercise programs and to overcome the one-size-fits-all approach (Barha et al., 2017b).

## Author Contributions

PM wrote the manuscript. PM, MT, and NM revised the manuscript.

### Conflict of Interest Statement

The authors declare that the research was conducted in the absence of any commercial or financial relationships that could be construed as a potential conflict of interest.
